# Supplementary material for: An ultraviolet-driven rescue pathway for oxidative stress to eye lens protein human gamma-D crystallin
Source: Commun Chem. 2024 Apr 10;7:81. doi: 10.1038/s42004-024-01163-w (PMC11006947; doi:10.1038/s42004-024-01163-w)
Supplement: Supplementary file 2 — Description of Additional Supplementary Files [file 42004_2024_1163_MOESM2_ESM.pdf]

Description of Additional Supplementary Files

File name: Supplementary Data 1

Description: fresh dataset, 83QL

File name: Supplementary Data 2

Description: aged dataset, 8BD0

File name: Supplementary Data 3

Description: light dataset, 8BPI
